# Supplementary material for: Fully Automated Production of (((S)-1-Carboxy-5-(6-([18F]fluoro)-2-methoxynicotinamido)pentyl)carbamoyl)-l-glutamic Acid ([18F]JK-PSMA-7)
Source: Pharmaceuticals (Basel). 2025 Jan 17;18(1):119. doi: 10.3390/ph18010119 (PMC11769433; doi:10.3390/ph18010119)
Supplement: Supplementary file 1 [file pharmaceuticals-18-00119-s001.zip › pharmaceuticals-3358454-supplementary.pdf]

Supporting Information

Article

Fully Automated Production of (((*S*)-1-Carboxy-5-(6-([18F]fluoro)-2-methoxynicotinamido)pentyl)carbamoyl)-L-glutamic Acid ([18F]JK-PSMA-7) †

Philipp Krapf [1], Thomas Wicher [1], Boris D. Zlatopolskiy [1,2], Johannes Ermert [1] and Bernd Neumaier [1,2,*]

[1] Forschungszentrum Jülich GmbH, Institute of Neuroscience and Medicine, Nuclear Chemistry (INM-5), Wilhelm-Johnen-Str., 52428 Jülich, Germany; p.krapf@fz-juelich.de (P.K.); t.wicher@rqs-ar.com (T.W.); j.ermert@fz-juelich.de (J.E.)

[2] Institute of Radiochemistry and Experimental Molecular Imaging, Faculty of Medicine and University Hospital Cologne, University of Cologne, Kerpener Str. 62, 50937 Cologne, Germany; boris.zlatopolskiy@uk-koeln.de (B.D.Z.)

* Correspondence: b.neumaier@fz-juelich.de; Tel.: +49-2461-614141

† This article is a revised and expanded version of a paper entitled "Fully automated and GMP-compliant synthesis of [18F]JK-PSMA-7 on a Trasis AllinOne module", which was presented at the 24th International Symposium on Radiopharmaceutical Sciences, iSRS 2022, Nantes, France, 29 May 2022–3 June 2022.

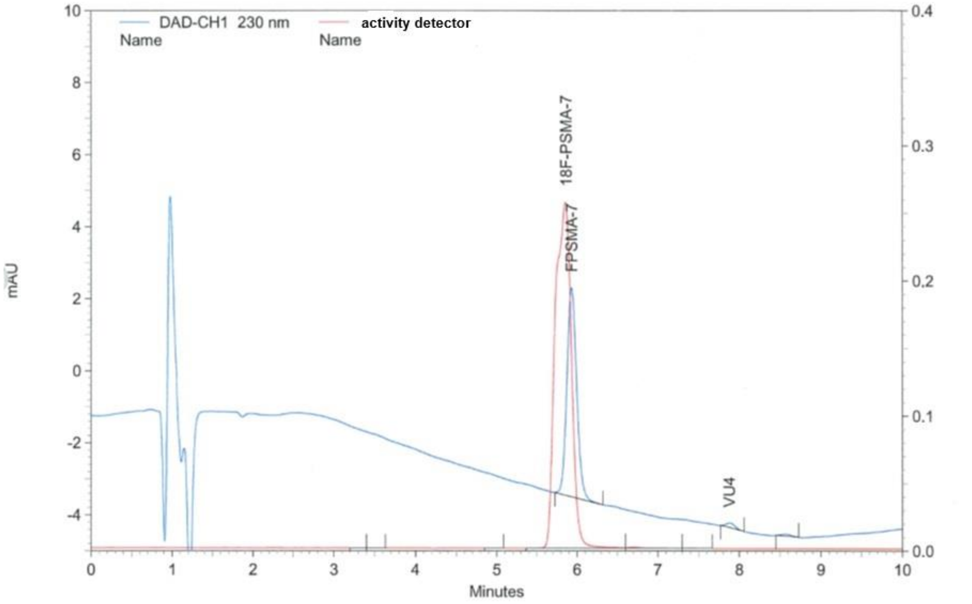

DAD-CH1 230 nm Results

Name	Retention Time	Area	Area %	conc. µg/mL
FPSMA-7	5.93	194931	96.7	1.88
VU4	7.88	4459	2.2	0.04
	8.56	2154	1.1	0.00
VUges		4459	2.2	0.04

Activity detector results

Name	Retention Time	Area Percent
18F-PSMA-7	5.87	99.5

Figure S1: HPL-Chromatogram of [^{18}F]JK-PSMA-7 and co-elution with JK-PSMA-7 standard.
